# Acute Traumatic Patellar Tendon Rupture at the Tibial Tuberosity Attachment without Avulsion Fracture

**DOI:** 10.1155/2017/2537028

**Published:** 2017-08-10

**Authors:** Shuichi Miyamoto, Makoto Otsuka, Fumio Hasue, Takayuki Fujiyoshi, Koushirou Kamiya, Hitoshi Kiuchi, Ken Ohara, Atsushi Yunde, Yasunori Toki, Tadashi Tanaka, Junichi Nakamura, Seiji Ohtori

**Affiliations:** ^1^Kimitsu Central Hospital, 1010 Sakurai, Kisarazu City, Chiba 292-8535, Japan; ^2^Graduate School of Medicine, Chiba University, 1-8-1 Inohana, Chuo-ku, Chiba City, Chiba 260-8677, Japan

## Abstract

Patellar tendon rupture in children is especially rare. The fact that the area of traumatic rupture has wide variations makes surgical treatment difficult. We present an 11-year-old boy with acute traumatic patellar tendon rupture at the tibial tuberosity attachment without avulsion fracture. Primary end-to-end repair and reinforcement using 1.5 mm stainless steel wires as a surgical strategy were undertaken. Early range of motion began with a functional knee brace and the reinforced stainless wire was removed 3 months after surgery. Knee function at the final follow-up was satisfactory. We suggest that this strategy may provide a useful option for surgical treatment.

## 1. Introduction

Although trauma around the knee joint is common and the extent of injury can vary widely in adolescents, quadriceps or patellar tendon tears are rare, occurring only about one-sixth as often as patellar fractures [1]. Patellar tendon ruptures are even rarer in childhood [2]. It generally is accepted that a healthy patellar tendon will not rupture without trauma. Previous reports show that ruptures can occur from the proximal end as a sleeve fracture of the patella to the distal end as an avulsion fracture of the tibial tuberosity [3–5]. These variations make surgical treatment difficult.

We report a case of a child who had acute traumatic patellar tendon rupture at the tibial tuberosity attachment without avulsion fracture. Treatment was performed with primary end-to-end repair and reinforcement with stainless steel wires. Knee function was satisfactory at the final follow-up.

## 2. Case Presentation

**Figure 1:**
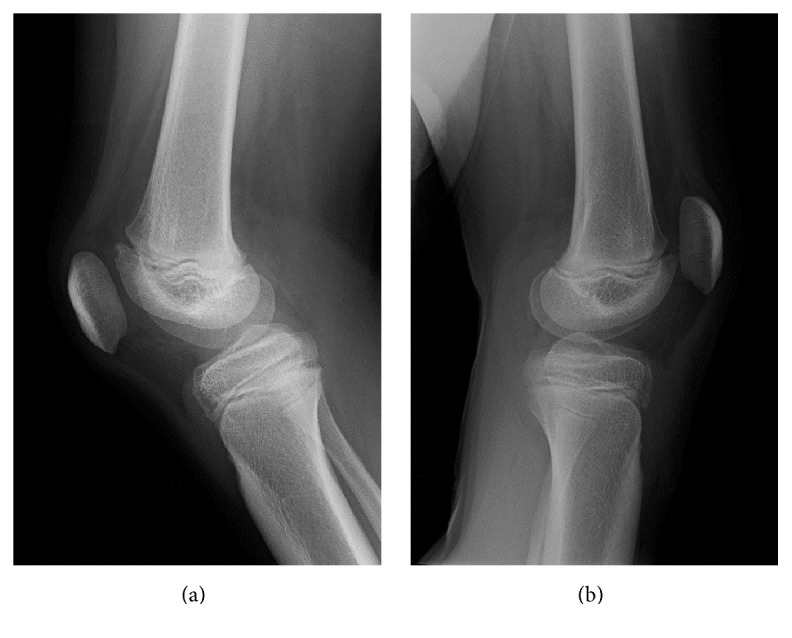
Preoperative lateral radiographs of the (a) right knee and (b) left knee.

**Figure 2:**
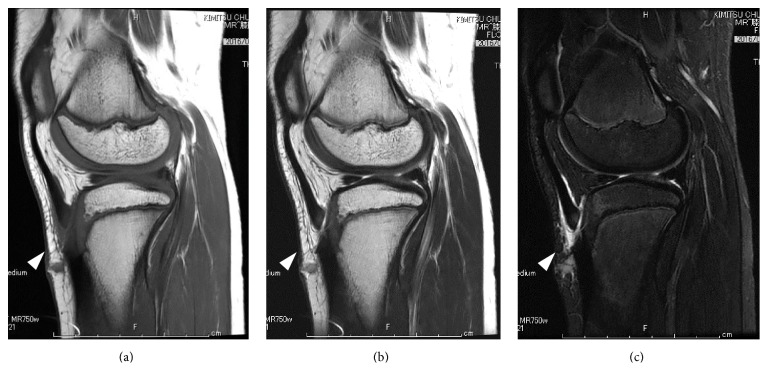
Preoperative lateral MRI of the left knee on (a) a T1-weighted image, (b) a T2-weighted image, and (c) a short T1 inversion recovery (STIR) image. The white arrowhead indicates a rupture of the patellar tendon.

Informed consent was obtained from the patient who took part in this report and from his parents. An 11-year-old boy was admitted to our hospital because of left-knee pain and bleeding from an incised wound after running into a glass door. Although he could not actively flex or extend his knee due to the pain and swelling, it was possible to slowly and passively flex. Physical examination revealed a transverse wound from 4 to 5 cm long on the tibial tuberosity. Using local anesthetics, we directly observed a complete tear of the patellar tendon. Lateral X-rays taken at 10 degrees of flexion revealed patella alta on the left side, which was evaluated using the Insall–Salvati method [6]. For right and left sides, the Insall–Salvati ratio was 1.2 and 1.7, respectively (Figure 1). Magnetic resonance imaging (MRI) demonstrated a rupture of the left patellar tendon at the tibial tuberosity attachment without a bone bruise or fracture (Figure 2). The boy's knee was immobilized in an extension brace.

**Figure 3:**
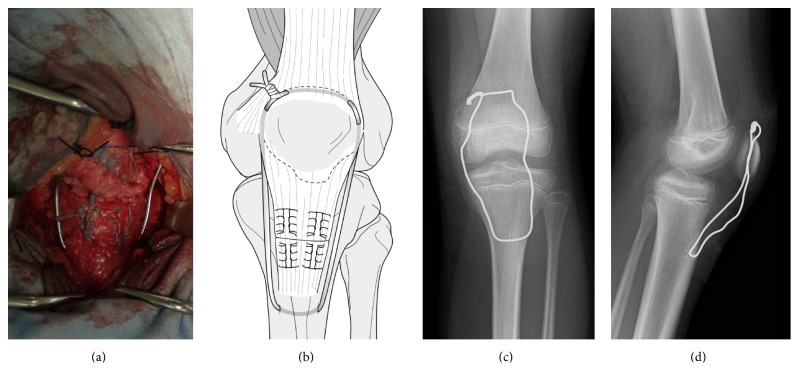
(a) Intraoperative view and (b) drawing of end-to-end repair and reinforcement with a wire cerclage. (c) Postoperative anteroposterior radiograph of the left knee. (d) Postoperative lateral radiograph of left knee.

Four days after injury, the patient underwent surgical treatment in a supine position under general anesthesia. A femoral tourniquet was applied to control bleeding. A transverse incision over the wound on the tibial tuberosity was performed. Intraoperatively the ends of the ruptured patellar tendon were explored and about 5 mm of patella tendon from the tibial tuberosity attachment remained. The ruptured ends were repaired with Number 2 Fiber wires (Arthrex Japan, Tokyo, Japan) by using the tendon repair technique described by Krackow et al. [7]. This repair was reinforced using a 1.5 mm stainless steel wire (Stryker, Tokyo, Japan), which was passed around the patella and through the drill hole in the proximal tibia (Figure 3).

The surgical wound healed without complications. The patient was placed in a functional Townsend knee brace (MEDX, Tokyo, Japan), and flexion was limited to a maximum of 90 degrees for 4 weeks postoperatively. The boy was allowed complete weight bearing as tolerated with the support of crutches and active rehabilitation based mainly on quadriceps stretching and knee exercises with limited range of motion. After 4 weeks postoperatively, the brace was adjusted to permit a gradual increase by 20 degrees of flexion every 2 weeks and removed 12 weeks postoperatively. Three months after surgery, the reinforced stainless steel wire was removed.

**Figure 4:**
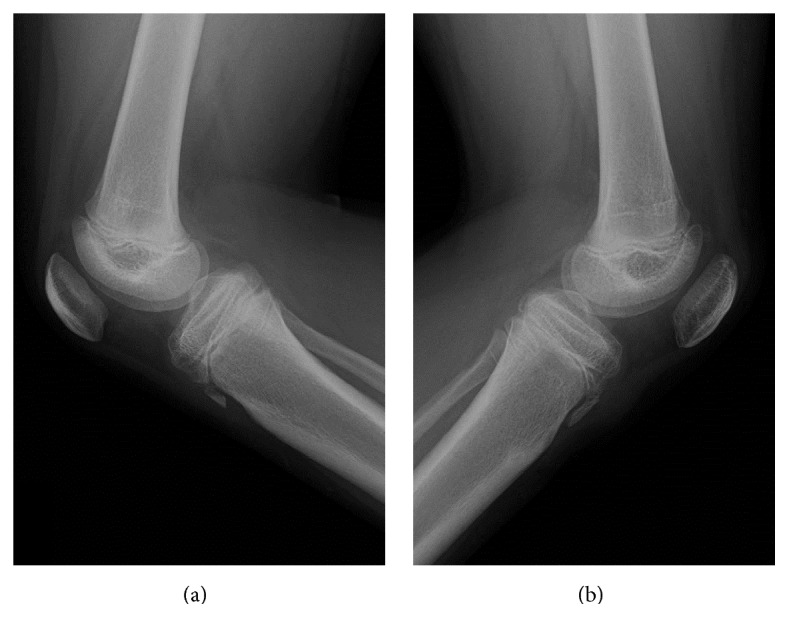
One year after surgery, lateral radiographs of the (a) right knee and (b) left knee.

**Figure 5:**
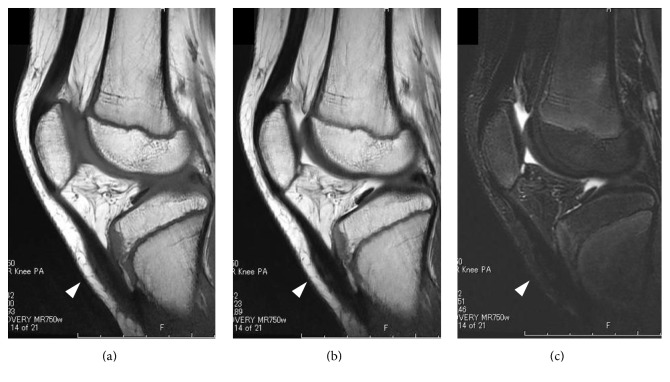
One year after surgery, lateral MRI of the left knee on (a) a T1-weighted image, (b) a T2-weighted image, and (c) a STIR image. The white arrowhead indicates the tendinous continuity of the patellar tendon.

At final follow-up one year postoperatively, the range of motion of the left knee was 0–120 degrees. Symptoms including pain and swelling of the knee were absent, and active flexion and extension were possible. Knee function was assessed using the Knee and Osteoarthritis Outcome Score for Children (KOOS-Child) subscales [8]. Pain was 100, symptoms scored 96, ADL was 100, Sport/Play was 93, and QOL was 100 on this scale. X-ray imaging showed the measurement of the Insall–Salvati ratio at 30 degrees of flexion was 1.2 for both knees (Figure 4). The MRI confirmed tendinous continuity between the ends of the tear (Figure 5).

## 3. Discussion

In the current study, we present a treatment of acute traumatic patellar tendon rupture at the tibial tuberosity attachment without avulsion fracture. Knee function at the final postoperative follow-up was favorable. Contrary to common closed patellar tendon ruptures previously reported, the area or severity of a patellar tendon rupture that occurred from an accident in this case might not be treated most effectively by standard surgical strategies.

The patellar tendon is the most central of the extensor mechanism, which includes the quadriceps muscles, their tendon, the patella, and the patellar tendon that attaches to the tibial tuberosity. Biomechanical studies of a normal patellar tendon showed that the force required to disrupt a patellar tendon is 17.5 times the body weight [9]. The usual mechanism of rupture is a knee flexion moment against a contracted or contracting quadriceps muscle [10]. Rupture of the patellar tendon means disruption of the extensor mechanism [11]. To date, few reports of acute extensor mechanism injuries distal to the patella in childhood have been reported [3–5, 12].

Acute patellar tendon ruptures require surgical treatment to recover the extensor mechanism, and early intervention improves functional rehabilitation. However, several surgical methods have been reported for different rupture severities. For full-tendon rupture, primary end-to-end sutures of each tendon with reinforcement by wire cerclage provide clinically acceptable function [13, 14]. Bushnell et al. [15] reported using a suture anchor technique in primary repair. In this report, restoration of knee function to 79% was clinically excellent, but the authors suggested the importance of a randomized trial comparing other options for repair.

Reinforcement frames using wire cerclage or augmentation are widely recognized as a treatment in both children and adults [1, 2, 14, 16]. In a biomechanical study of various augmentations, a comparative experiment was performed to examine the difference between hamstring autograft augmentation and repair using three transpatellar sutures [17] augmented with a Number 5 Ethibond suture or a 2.0 Dall-Miles cable and repair using two transpatellar Krackow sutures [18]. A previous report of patellar tendon rupture in childhood showed good clinical knee function following end-to end repair, reinforcement with cerclage wires, and fresh-frozen Achilles tendon augmentation [2]. These results suggest that reinforcement by augmentation is necessary. However, because patellar tendon rupture is so uncommon and the patient sample in these reports was very small, comparison between surgical techniques is extremely difficult. To do so, it is vital for the surgeon to identify the area of rupture and the mechanism of trauma through a careful history prior to surgery [16]. This will help the surgeon to explore various surgical options.

Postoperative management and rehabilitation have important implications for function of the knee joint. Previous reports about patellar tendon repair have recommended early mobilization to prevent both amyotrophy of quadriceps and joint contracture [19, 20]. If patient compliance, stability, and durability of the repair during surgery are acceptable, we propose that rigid immobilization after surgery is not necessary. In the current report, early range of motion began one day after surgery with a functional knee brace limiting flexion to 90 degrees for 4 weeks. Because tensile stress was not detected in the repaired ends of the tendon during intraoperative passive 90 degrees of flexion, additional flexion gradually was permitted every 2 weeks postoperatively. Knee function at the final follow-up was satisfactory.

## 4. Conclusion

We present a case of acute traumatic patellar tendon rupture at the tibial tuberosity attachment without avulsion fracture. With primary end-to-end repair and reinforcement using stainless wires, the intervention of early mobilization may be useful and effective surgical treatment.
